# Marked increase in severe neurological disorders after nitrous oxide abuse: a retrospective study in the Greater Paris area

**DOI:** 10.1007/s00415-024-12264-w

**Published:** 2024-03-13

**Authors:** Yachar Dawudi, Loris Azoyan, Thomas D. E. Broucker, Thierry Gendre, Amal Miloudi, Andoni Echaniz-Laguna, Julie Mazoyer, Adrien Zanin, Nathalie Kubis, Anne-Laure Dubessy, Lucas Gorza, Haifa Ben Nasr, Weniko Caré, Thibaut d’Izarny-Gargas, Aude Formoso, Ana-Maria Vilcu, Mickael Bonnan

**Affiliations:** 1 Neurology Department, Centre Hospitalier de Saint-Denis, 2 Rue du Dr Delafontaine, 93200 Saint-Denis, France; 2 Institut Pierre Louis d’épidémiologie et de Santé Publique (IPLESP UMRS 1136, Sorbonne Université INSERM, Paris, France; 3 Neurology Department, Hôpital Henri-Mondor, 1 Rue Gustave Eiffel, 94000 Créteil, France; 4 Neurology Department, Centre Hospitalier Intercommunal Robert Ballanger, Bd Robert Ballanger, 93600 Aulnay-Sous-Bois, France; 5 Neurology Department, Hôpital Bicêtre, 78 Rue du Général Leclerc, 94270 Le Kremlin-Bicêtre, France; 6 Neurology Department, Hôpital Avicenne, 125 Route de Stalingrad, 93009 Bobigny, France; 7 Clinical Physiology Department, Hôpital Lariboisière, 2 Rue Ambroise Paré, 75010 Paris, France; 8 Neurology Department, Hôpital Saint-Antoine, 184 Rue du Faubourg Saint-Antoine, 75012 Paris, France; 9 Neurology Department, Hôpital Foch, 40 Rue Worth, 92150 Suresnes, France; 10 Neurology Department, Centre Hospitalier Sud Francilien, 40 Avenue Serge Dassault, 91100 Corbeil-Essonnes, France; 11 Department of Internal Medicine, Bégin Military Teaching Hospital, Saint-Mandé, France; 12 Paris Poison Control Center, Toxicology Federation (FeTox), Hôpital Fernand Widal, AP-HP Paris, France; 13 Paris Cardiovascular Research Center, INSERM U970, Paris, France; 14 Neurology Department, Hôpital de La Pitié Salpêtrière, 47-83 Bd de L’Hôpital, 75013 Paris, France

**Keywords:** Epidemiology, Nitrous oxyde abuse, Myelopathy, Peripheral neuropathy

## Abstract

**Background:**

Recreational nitrous oxide (N_2_O) use has become more widespread worldwide, leading to an increase in myelopathies and peripheral neuropathies. The aim of this study was to describe clinical and socioeconomical characteristics of severe N_2_O-induced (NI) neurological disorders (NI-NDs), to determine its incidence in the Greater Paris area and to compare it with that of similar inflammatory neurological disorders.

**Methods:**

We performed a retrospective multicentric cohort study of all adult patients with severe NI-NDs in the neurology and general internal medicine departments of the Greater Paris area from 2018 to 2021. The incidence was compared with that of non-NI-myelitis and Guillain–Barré syndrome (GBS) using a sample of 91,000 hospitalized patients sourced from health insurance data.

**Results:**

Among 181 patients, 25% had myelopathy, 37% had peripheral neuropathy and 38% had mixed disease. Most were aged between 20 and 25 years, lived in socially disadvantaged urban areas, and exhibited high rates of unemployment (37%). The incidence of NI-NDs increased during 2020 and reached a peak mid-2021. The 2021 incidence in 20–25-year-olds was 6.15 [4.72; 8.24] per 100,000 persons for NI-myelopathy and 7.48 [5.59; 9.37] for NI-peripheral neuropathy. This was significantly higher than for non-NI-myelitis (0.35 [0.02; 2.00]) and GBS (2.47 [0.64; 4.30]). The incidence of NI-NDs was two to three times higher in the most socially disadvantaged areas.

**Conclusion:**

The recent increase in recreational N_2_O use has led to a rise in the incidence of severe NI-NDs, particularly in young adults with low socioeconomic status for whom NI-NDs strongly outweigh similar neurological disorders.

**Supplementary Information:**

The online version contains supplementary material available at 10.1007/s00415-024-12264-w.

## Introduction

In recent years, a sharp rise in nitrous oxide (N_2_O) use for recreational purposes has been observed worldwide [1–3]. Due to the inactivation of the cobalt ion responsible for the loss of vitamin B12 activity [4], N_2_O inhalation can lead to central and peripheral neurological disorders, i.e. myelopathy and peripheral neuropathy. Consequently, the incidence of these complications has increased dramatically to become a major public health concern [5–7].

The clinical presentation may mimic differential diagnoses which necessitate prompt and aggressive treatment approaches, such as the administration of intravenous immunoglobulins or corticosteroids. Furthermore, as the incidence of severe N_2_O-induced (NI) neurological disorders (NI-NDs) increases, so does the risk of confusing NI-NDs with its differential diagnoses, potentially leading to therapeutic errors. Although several small series are available, the incidence of NI-NDs has only been captured indirectly through spontaneous drug safety notifications [8]. We investigated the incidence and characteristics of severe NI-NDs among adults in the Greater Paris area. We assessed the incidence of central and peripheral NI-NDs and compared it to that of the main severe non-NI neurological disorder (non-NI-NDs) differential diagnoses: myelitis and Guillain–Barré syndrome (GBS).

## Methods

To estimate the incidence of severe NI-NDs in the Greater Paris area, we established a retrospective multicentric cohort of adult patients with severe NI-NDs from January 1, 2018, to December 31, 2021. We contacted all neurology and general internal medicine departments in the region. The severe NI-NDs considered were hospital-attended N_2_O-induced myelopathy and peripheral neuropathy. Patients were included in the NI-NDs cohort if they were over 18 years old and presented neurological symptoms during the target period. Symptoms had to be related to N_2_O consumption with no alternative diagnosis and taken care of/sought hospital care in neurology or internal medicine. Exclusion criteria included being under legal protection (guardianship, curatorship) at the time of hospital consultation. Demographic and paraclinical data were collected by the referring physician using a standardized grid including demographic, clinical, biological and therapeutic data, as well as follow-up data when available (Supplementary Table S1: Survey Form). Demographic data for the Greater Paris area were extracted via government agencies (Details in Supplementary data: Research Protocol). The study received ethics committee approval from Hôpital Delafontaine, Saint-Denis. The incidence of NI-NDs was compared with similar differential diagnoses, which were hospital-attended non-N_2_O-induced myelitis and Guillain–Barré syndrome (GBS), which is the most common form of severe peripheral neuropathy. To estimate the incidence rate of severe non-NI-NDs, we used hospitalization data from the *Échantillon Généraliste de Bénéficiaires* (EGB), a 1/97th permanent and representative (in terms of age, sex, and medical expenses) sample of the French Health Insurance database [9] (consisting of 91,000 inpatients). We identified all adult patients hospitalized for myelitis or GBS from January 1, 2011, to December 31, 2019.

Disorders were ascertained based on disease-specific ICD-10 codes recorded as main or related diagnosis (G373 for acute transverse myelitis in demyelinating disease of central nervous system and G610 for GBS). Inclusion criteria for the EGB cohort were being aged 18 years or over on January 1 of each year and having at least 2 years of presence in the EGB database prior to January 1 of each year, with exclusion criteria including presenting any of the studied diseases in the 2 years preceding January 1 of each year.

Considering that severe NI-NDs were rare before 2019 [10] and assuming stable incidence of inflammatory neurological disorders over time, we deemed it appropriate to compare the overall and age-group pre-2019 incidence rates of GBS and myelitis with the 2018–2021 incidence rates of NI-neuropathy and NI-myelopathy, respectively.

We estimated 95% binomial proportion confidence intervals. Non-overlapping 95% CI indicate significant differences between the incidence rates. For the age analysis, age was split into 5-year categories. Detailed information on the research protocol is available in Supplementary data. We followed the recommendations of the RECORD declaration (REporting of Conducted Studies using Observational Routinely-collected health Data).

## Results

**Fig. 1:**
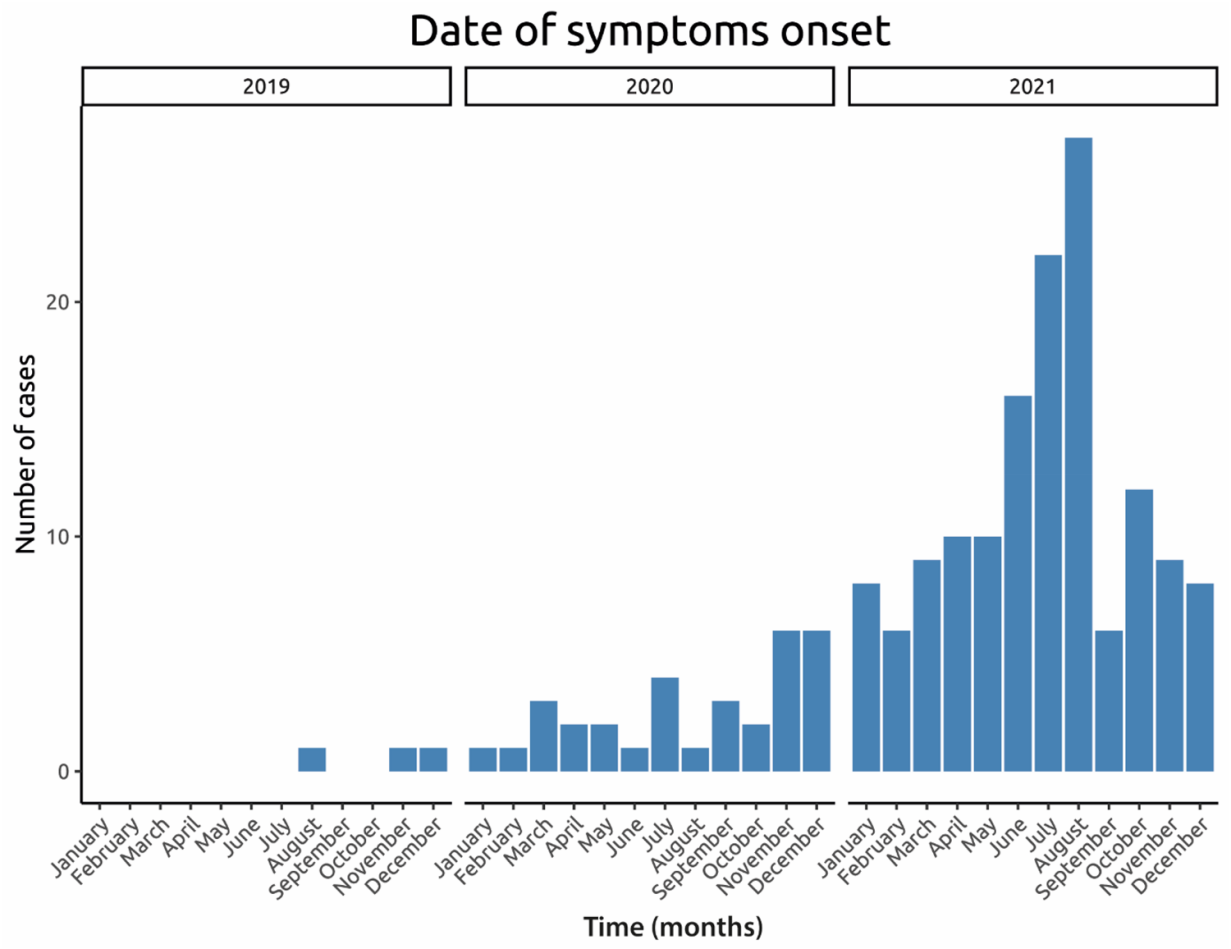
Incidence of N_2_O-related neurological disorders in the Greater Paris area during the period 2019–2021. The first cases reported date from 2019, then an increase in cases is observed with a peak in 2021

Out of the 81 units contacted, 78 (96%) participated in the retrospective study (Supplementary Fig. S1) and reported a total of 181 patients with NI-NDs. One hundred and sixty-nine patients (95%) came from a neurology department and 143/178 (80%) were hospitalized. Average daily consumption was 2 carboys (equivalent to 1200 g of N_2_O). Median duration between the beginning of N_2_O consumption and the onset of symptoms was 6 (IQR 2–12) months. No cases were detected before the end of 2019. The incidence then increased sharply in 2020, with a maximum of 143/178 (80%) cases in 2021 (Fig. 1).

One hundred and seven patients (60%) were aged between 20 and 25 years, with no previous medical history in 169/178 (95%) of cases, 44/181 (25%) had myelopathy, 66/181 (37%) had peripheral neuropathy and 68/181 (38%) had mixed disease. Sensorimotor disorders were the main cause of disability, with an acute Rankin score ≥ 4 in 42/128 (33%) cases with available data. Data on clinical evolution after 1 month were available for 64 patients, with full recovery reported only in 5/64 (8%). One hundred sixty-eight patients (96%) were treated with Vitamin B12. Additionally, 11% (18/161) self-medicated with Vitamin B12 before hospitalization without altering their consumption habits.

The main demographic, clinical and biological data are shown in Table 1, with additional information in Supplementary Table S2.

**Table 1:** Main demographics, clinical and biological results

Characteristics	*N* = 181^‡^
Age in years	23.1 (4.7)
Men	96/178 (54%)
Situation	
Student	24/167 (14%)
Unemployed	61/167 (37%)
Worker	82/167 (49%)
Smoker	99/169 (59%)
Cannabis use	36/147 (24%)
Frequent alcohol use	25/166 (15%)
N_2_O consumption	
Festive	97/154 (63%)
Mixed	32/154 (21%)
Solitary	25/154 (16%)
Mode of consumption	
Carboy	91/127 (72%)
Cartridge	57/127 (45%)
Motor deficiency	127/177 (72%)
Sensory deficiency	175/178 (98%)
Sphincter dysfunction	33/176 (19%)
Gait disorder	144/172 (84%)
Neurological damage	
Mixed	68/178 (38%)
Myelopathy	44/178 (25%)
Neuropathy	66/178 (37%)
MRI lesion	95/154 (62%)
MRI extension (number of vertebrae levels)	5 (4, 7)
Electromyography	
Axonal lesion	83/142 (58%)
Demyelinating lesion	9/142 (6%)
Mixed lesion	28/142 (20%)
Abnormal CSF analysis	12/67 (18%)
Average hemoglobin, g/dL	13.28 (1.65)
Average MCV, fL	93 (9)
Abnormal vitamin B12	63/133 (47%)
High MMA and/or Hcy	113/118 (96%)

Mean (standard deviation) or *n*/*N* (%)

Abbreviations: *CSF* cerebrospinal fluid, *Hcy* homocysteine, *MCV* mean corpuscular volume, *MMA* methylmalonic acid, *MRI* magnetic resonance imaging

^‡^Three centers each reported one case, but without giving any further details

In the Greater Paris area, there were 60/181 (33%) cases in Paris, 109/181 (61%) in the inner suburbs, and only 29/181 (6%) in the outer ones (Supplementary Fig. S2). Outside Paris, 62/109 (57%) cases were reported in Seine-Saint-Denis, while some more privileged administrative counties reported none.

**Fig. 2:**
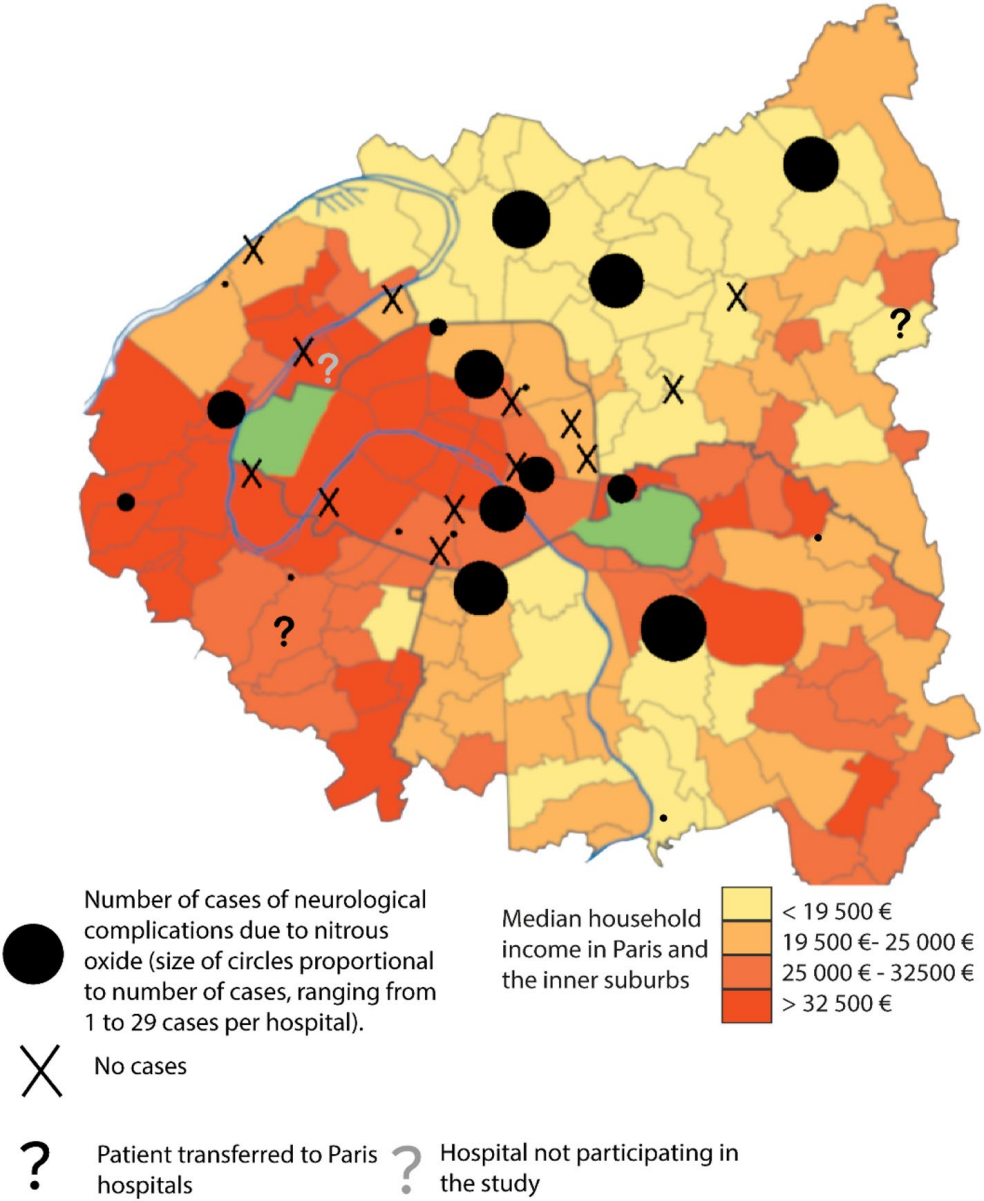
Distribution of cases by household income in Paris and inner suburbs. The majority of cases are located in lower income areas of the inner suburbs

The unemployment rate was higher in cohort patients (61/167; 37%) than in the Greater Paris area population (8%; *p* < 0.001), and workers often had low-income jobs. The most affected administrative counties were also the most socially disadvantaged ones. Seine-Saint-Denis, the administrative county with the highest incidence of NI-NDs, has the lowest average incomes (Fig. 2) and 19% of its inhabitants received welfare for the unemployed versus 10% in the whole Greater Paris area [11,12].

**Fig. 3:**
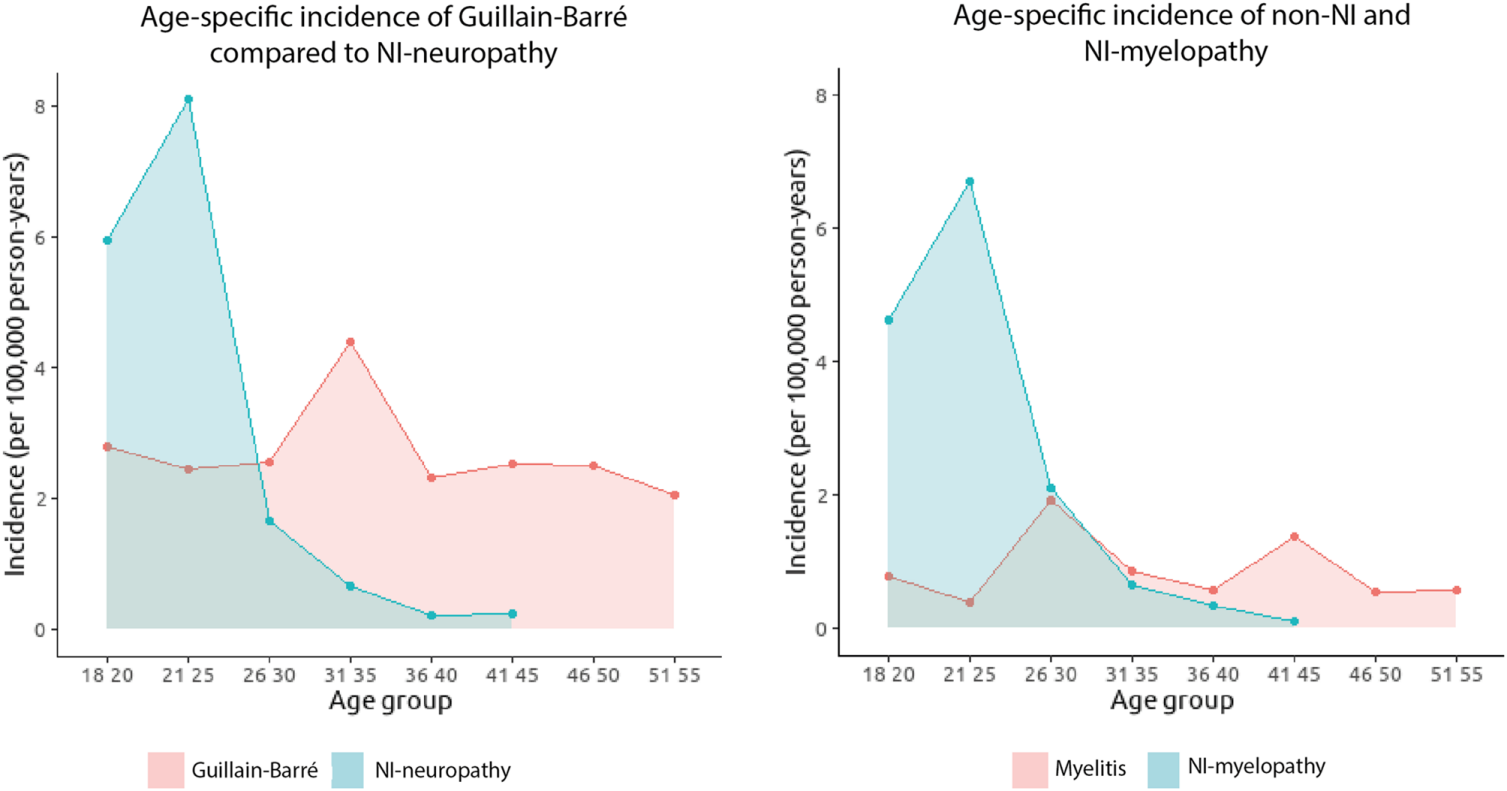
Age-specific incidence of NI and non-NI neurological disorders. There is a significantly higher incidence of neurological complications in individuals under the age of 25 following N_2_O intoxication compared with similar disorders: NI peripheral neuropathy and GBS (left panel), and NI/non-NI myelopathy (right panel). Abbreviation: *GBS* Guillain–Barré syndrome, *NI* N_2_O-induced

In the Greater Paris area, the incidence of NI-myelopathy and non-NI myelitis was 0.75 [95% CI 0.6; 0.91] and 0.66 [95% CI 0.41; 0.92] cases per 100,000 person-years, respectively. The incidence of NI-neuropathy and GBS was 0.84 [95% CI 0.67; 1.00] and 3.77 [95% CI 3.17; 4.38] cases per 100,000 person-years, respectively. Among the 20–25 years old individuals, the incidence of NI-myelopathy was 6.15 [95% CI 4.72; 8.24] cases per 100,000 person-years, which is significantly higher than that of non-NI myelitis estimated at 0.35 [95% CI 0.02; 2.00] cases per 100,000 person-years (Fig. 3). In this same age group, the incidence of NI-neuropathy was 7.48 [95% CI 5.59; 9.37] cases per 100,000 person-years, which was significantly higher than that of GBS with 2.47 [95% CI 0.64; 4.30] cases per 100,000 person-years. In Seine-Saint-Denis, the rate of NI-myelopathy and neuropathy in individuals 20–25 years old individuals was 16.64 [8.96; 24.33] and 20.34 [11.84; 28.84], respectively, which was significantly higher than in the whole Greater Paris area.

## Discussion

This multicenter study investigated a large cohort of severe NI-NDs to estimate its incidence, and compared it with similar neurological disorders in the same population. The estimated incidence of NI-NDs was significantly higher than the overall incidence of similar neurological diseases in young people, due to a sharp increase in incidence after 2020 that peaked in 2021. Most patients were young people from socially disadvantaged areas. Complications are severe with motor deficits and significant disability, and complete early recovery was rare.

Since no specific diagnosis code was available to compute the incidence of NI-NDs, our method of collecting individual cases was the only possible method to estimate the incidence of these complications.

The main limitation of our study is that we compared incidence obtained from cases collection to incidence obtained from administrative health database. However, we consider this approach acceptable as selecting only severe NI-NDs could only lower the estimated incidence and the true gap with the incidence of non-NI-NDs could be even greater. The likelihood of having missed any hospitalized cases seems low due by two factors: the almost complete participation of all centers in Greater Paris and a recall bias considered limited.

The latter is attributed to a relatively short study period (4 years) and particularly striking cases due to the novelty of the disease, the severity of the conditions in young patients without medical history and their extraordinarily high consumption of N_2_O.

The low probability of having missed hospitalized cases is primarily supported by two factors: the near-complete participation of hospital centers in the Paris area and a low memory bias. The latter is attributed to the limited duration of the study, which spans four years, thereby facilitating the recall of information. Moreover, memory retention is enhanced by the unusual nature of the cases observed, characterized by the novelty of the disease, the severity of symptoms in young patients without medical history, and their exceptionally high level of consumption.

A further limitation is a possible border effect as geographical localisation of cases is based on hospitalisations and patients might seek hospital care beyond their nearest facility. However, as individual case data were compared with ecological data on the income levels of the areas, conclusions are inherently broad. Precise individual level conclusions are not possible, we can generally infer that lower-income areas have a higher incidence of cases. Furthermore, the observed higher case numbers in Paris, despite its higher income levels, may be attributed to its greater hospital density. It is interesting to note that a stronger concentration of cases is observed in areas of Paris adjacent to the Parisian suburbs where income is lowest, which could be explained by this border effect.

N_2_O was the 8th most consumed drug globally in 2017, particularly popular among medical students and the nightclub scene [13–15]. During the same period and in the same area, medical students reported an average consumption of 2 cartridges per year, which is equivalent to 16 g of N_2_O annually, representing a quantity 27,000 times lower than that the mean consumption observed in our study [16]. Moreover, medical students did not manifest any severe neurological complications. This points to a dose-dependent effect of N_2_O consumption on the onset of neurological disorders as suggested in other studies [17].

In line with DSM-V criteria [18], N_2_O consumption in our cohort was characteristic of an addictive disorder, in contrast to previous debates on the addictive potential of N_2_O [15]. Moreover, the psychological profile of these users indicates a marked psychiatric vulnerability [19]. Despite consumption dating back to the eighteenth century, reported cases of NI-NDs were exceptional until recently [10]. A systematic review of literature up to 2018 only found a hundred of patients, published solely in case reports [10]. The recent increase could be attributed to both more frequent consumption and higher intake, a trend that, despite methodological differences in studies, indicates a substantial rise in reported cases that likely surpasses the impact of potential biases. The incidence rates reported here relate only to severe NI-NDs, as we focused on patients suffering from signs severe enough to initiate a hospital diagnosis and care. However, as 2–8% of N_2_O users may experience minor sensory symptoms [17], therefore the overall rate of NI-NDs is probably far higher.

While this study was restricted to the Greater Paris area, which comprises 20% of the French population (12.4 million inhabitants), N_2_O consumption has become a worldwide practice [6–8,20]. In France, both COVID-19 and lockdown may have contributed to the increased consumption [8,21], but there was already a global trend of increased use before the pandemic in other countries [22,23]. Globally, the escalation in N_2_O consumption, as well as the reported increase in complications, dates back to around 2018, coinciding with the introduction of N_2_O carboys on the market^23,24^. These carboys contain on average 100 times the dose contained in a standard cartridge and their ease of use may have facilitated higher consumption, which could be another factor contributing to the increase in N_2_O complications.

## Conclusion and relevance

High levels of N_2_O intake may cause severe NI-NDs with disability and uncertain recovery. In 2021, incidence of NI-NDs was significantly higher than those of myelitis and GB. Most affected patients were young people from socially disadvantaged areas. Our findings not only point to a medical concern but also shed light on underlying socioeconomic factors and potential addictive behaviors associated with N_2_O use. These results indicate a marked shift in consumption patterns towards larger and harmful doses. Given the global prevalence of N_2_O use, this might signal a growing public health issue extending beyond France’s borders. Education campaigns and comprehensive prevention strategies would be crucial to address this concerning trend.

### Supplementary Information

Below is the link to the electronic supplementary material.

Supplementary file1 (DOCX 1195 KB)

## Data Availability

Data not presented in the article are available in the supplements. If more data are necessary, please contact us by mail.
