# Supplementary material for: Marked increase in severe neurological disorders after nitrous oxide abuse: a retrospective study in the Greater Paris area
Source: J Neurol. 2024 Mar 13;271(6):3340–6. doi: 10.1007/s00415-024-12264-w (PMC11136741; doi:10.1007/s00415-024-12264-w)

**Supplementary data**

#

# Research protocol

This study was designed in the context of a growing number of young patients seeking medical advice due to acute onset of severe nitrous oxide (N_2_O)-induced neurological disease (NI-NDs). It aimed to evaluate the incidence of severe NI-NDs in the Greater Paris area and compare it with the incidence of similar differential diagnoses. The severe NI-NDs considered were hospital-attended N_2_O-induced myelopathy and peripheral neuropathy, while the similar differential diagnoses considered were hospital-attended non-N_2_O induced myelitis and Guillain-Barré syndrome (GBS), which is the most common form of severe peripheral neuropathy.

**A. Collection of data and design of cohorts**

In France, no available health data sources allow the accurate identification of NI-NDs cases. Indeed, experience has shown that these recently emerged conditions are difficult to identify in hospital databases due to the complex and often low quality of ICD-10 diagnosis coding, and that they are grossly under-declared in toxicology databases. Thus, to compute the incidence of central and peripheral NI-NDs in the Greater Paris area, we performed a retrospective multicentric study of adult patients hospital-attended for neurological phenomena related to N_2_O consumption (Nitrous oxide cohort below).

To calculate the incidence of severe non-NI myelitis and non-NI GBS in the population residing in Metropolitan Paris, we used data on hospital stays recorded in the *L’Échantillon Généraliste de Bénéficiaires* (EGB) database, which is a representative sample of the National French Health Insurance system^1,2^ (see below).

**1. Nitrous Oxide cohort**

**Cohort description and data collection**

We constituted a multicenter retrospective cohort of adult patients who consulted for neurological phenomena related to N_2_O consumption in a neurology or internal medicine department of hospitals in the Greater Paris area, from January 1, 2018, to December 31, 2021. The Greater Paris area is home to 12.5 million people, or 20% of the French population. It comprises eight administrative counties: Paris (75), Seine-et-Marne (77), Yvelines (78), Essonne (91), Hauts-de-Seine (92), Seine-Saint-Denis (93), and Val-de-Marne (94). All hospitals in the Greater Paris area are listed on the regional health agency website^3^.

All departments capable of admitting this type of patient, i.e. neurology or internal medicine, were contacted by email or telephone. We managed to contact one physician in each center. Non-participating centers are those that clearly refused to take part in the study or did not reply after five reminders or more. The data were collected retrospectively by the physician in charge of the patient, and were anonymously collected by the authors (YD, LA).

Anonymous data were collected using a standardized grid (Supplementary data 1) that included non-identifying demographic information, pattern of drug use, clinical signs, main biological tests in blood and cerebrospinal fluid, magnetic resonance imaging (MRI) and electromyographic (EMG) data, and clinical outcome, when available. In addition, dates of onset of neurological signs and hospitalization (or consultation) due to N_2_O consumption were collected and were rounded to the month to preserve anonymity. Data relating to the period prior to hospitalization were collected from patients' self-reports.

By using a standardized grid and requiring additional examinations such as MRI and EMG to reveal the damage, we ensured that the condition was genuine. By considering only patients cared for in hospital, we ensured that the condition was of a certain severity. This allowed us to make comparisons with other diseases of comparable severity, such as non-NI myelitis and GBS.

**Patient inclusion criteria**

- Presenting with neurological phenomena related to N_2_O consumption in the Neurology or Internal Medicine departments of a hospital in Metropolitan Paris.
- Age ≥18 years at time of hospital referral.
- Having no alternative diagnosis other than N_2_O intoxication at the hospital consultation.
- Having suffered from a neurological disorder during the target period (January 1, 2018, to December 31, 2021).

**Exclusion criteria**

- Being under legal protection (guardianship, curatorship) at the time of hospital consultation.
- Refused to participate after receiving study information notice

**2. EGB cohort**

**Database description**

*L’Échantillon généraliste de bénéficiaires (EGB)* is a 1/97^th^ permanent sample of the SNDS (Système National des Données de Santé), the French health insurance database which covers 99% of the French population^1,2^. It contains anonymous socio-demographic and medical information on more than 700,000 individuals representative of the French population in terms of age, sex, and medical expenses. Information includes individuals prescribed medication dispensed in community pharmacies, hospital admissions (including diagnoses of hospital stays using the International Classification of Diseases – 10^th^ Version, ICD-10), and long-term diseases (LTD) giving entitlement to 100% health insurance coverage. Individuals included in the EGB database are followed up for up to 20 years, whether or not they have received any healthcare reimbursements. Thus, the database can be used for longitudinal studies and to reconstruct patients' care pathways over a long period, whether in the community or in hospital.

**Study population** **and study period**

This retrospective study was conducted between January 1, 2011 and December 31, 2019. The study population concerned all individuals included in the EGB database and residing in Metropolitan Paris, alive and 18 years of age or older, and having at least two years of presence in the EGB database on January 1 of each year of the study period.

**Inclusion criteria**

- Being aged 18 years or over on January 1 of each year
- Having at least two years of presence in the EGB database prior to January 1 of each year

**Exclusion criteria**

- Presenting any of the studied diseases in the two years preceding the January 1 of each year

**Identification of incident cases of severe myelitis and Guillain-Barré syndrome**

To identify adult patients suffering from inaugural myelitis related or not with multiple sclerosis or neuromyelitis optica spectrum disorder and GBS in Metropolitan Paris, all hospitalizations during the study period with a disease-specific ICD-10 code as a principal or related diagnosis were extracted from the EGB database. The ICD-10 codes considered were:

- G373: Acute transverse myelitis in demyelinating disease of central nervous system
- G610: Guillain-Barré syndrome

Since only the year of birth was available in the EGB database, patient age was calculated as the difference between the year of hospitalization and the year of birth.

To avoid misclassification of prevalent cases as incident cases, only the first hospitalization episode during the study period was considered for each disease. These patients were no longer counted in the analyses for the years after the incident episode. Patients with a history of hospitalizations for myelitis or GBS, and those registered with an LTD that can lead to myelitis such as multiple sclerosis in the two years preceding the year of the incident episode were considered prevalent cases and were excluded.

NI-NDs were very rare before 2019^4^. Thus, we assume that incident cases of myelitis and GBS identified in the EGB database during the study period were not induced by N_2_O consumption (non-NI).

National Institute for Health and Medical Research (INSERM) agreement for the research protocol was given on June 14, 2023. Neither approval by an ethics committee nor a request from national commissions for individual data protection were required according to French law, to access the restricted database of anonymously recorded data. Access to the EGB database is possible only through a secure connection to a specific server. Data are accessible online and were analyzed by the software SAS Enterprise Guide version 4.3 (Copyright ©2006–2010, SAS Institute Inc., Cary, NC, USA).

**3. Data on demographics**

The unemployment rate, the rate of the ‘Revenu de Solidarité Active’, which is social benefit given to unemployed persons), and median income in the Greater Paris area were obtained from publicly available data (*Direction de la recherche, des études, de l'évaluation et des statistiques*^5^, DRESS) and The *Institut national de la statistique et des études économiques*^6^*,* (INSEE). Smoking and cannabis consumption data were also obtained from public databases (*Santé Publique France*^7^*,* and the *Observatoire Français des Drogues et des Tendances Addictive*^8^*,* OFDT).

**B. Statistical analysis**

Clinical and demographic descriptive variables are given as median [inter quartile range], mean (SD) or n/N (%). We assumed a constant incidence of inflammatory neurological diseases (GBS and myelitis) over the study period. The incidence rates of NI-myelopathy and NI-neuropathy were estimated per year and over the entire period from January 1, 2018, to December 31, 2021, overall, by department, and by age group. The incidence rates of non-NI myelitis and non-NI-neuropathy were estimated over the entire period from January 1, 2011 to January 1, 2019, overall and by age group.

For each disorder, the yearly incidence rate was estimated by dividing the number of cases by the number of individuals at risk at the beginning of each year, and was expressed as number of cases per 100,000 inhabitants. The incidence rate over the study period was estimated as the number of cases over the entire study period divided by the number of person-years at risk over the study period, and was expressed as number of cases per 100,000 person-years. The 95% confidence intervals (95%CI) were estimated using normal approximation when there were at least five cases, and binomial distribution otherwise. Non-overlapping 95%CI indicate significant differences between the incidence rates. For the age analysis, age was split into 5-year categories.

Comparisons were made with Student's T test for mean comparison and the Chi2 test for percentage comparison. Descriptive analyses were conducted in R, version 4.2.3 (R Foundation).

**References**

1. Scailteux LM, Droitcourt C, Balusson F, et al. French administrative health care database (SNDS): The value of its enrichment. Therapies. 2019;74(2):215-223. doi:10.1016/j.therap.2018.09.072

2. De Roquefeuil L, Studer A, Neumann A, Merlière Y. L’échantillon généraliste de bénéficiaires : représentativité, portée et limites: Prat Organ Soins. 2009;Vol. 40(3):213-223. doi:10.3917/pos.403.0213

3. Agence régionale de santé | Agir pour la santé de tous. Accessed July 18, 2023. https://www.ars.sante.fr/

4. Caré W, Dufayet L, Piot MA, et al. Toxicités aiguës et chroniques associées à l’usage et au mésusage du protoxyde d’azote : mise au point. Rev Médecine Interne. 2022;43(3):170-177. doi:10.1016/j.revmed.2021.10.008

5. Accueil | Direction de la recherche, des études, de l’évaluation et des statistiques. Accessed November 1, 2023. https://drees.solidarites-sante.gouv.fr/

6. Accueil - Insee - Institut national de la statistique et des études économiques. Accessed November 1, 2023. https://www.insee.fr/fr/accueil

7. Accueil - Santé publique France. Accessed November 1, 2023. https://www.santepubliquefrance.fr/

8. OFDT - Observatoire français des drogues et des tendances addictives en France - Données, études, enquêtes, observations. Accessed November 1, 2023. https://www.ofdt.fr/

# Supplementary Tables

**
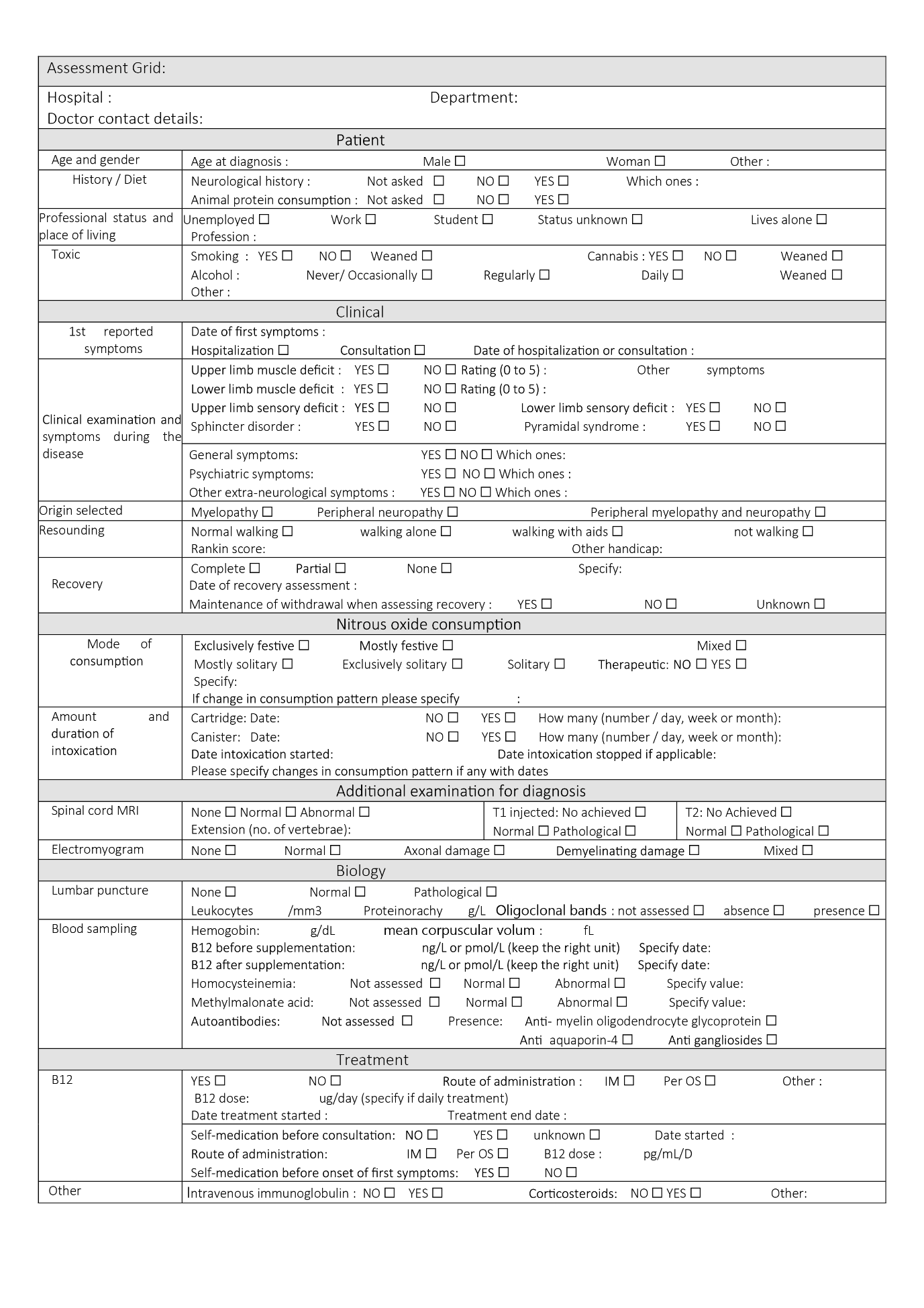
Table S1. Survey form: standardized grid for anonymous data collection**

# Table S2. Additional data.

| **Characteristics** | N = 181^1^ |
| --- | --- |
| **Unit:** *Neurology* | 169/178 (95%) |
| *Internal medicine* | 9/178 (5%) |
| **Neurological history** | 1/178 (0.6%) |
| **Other history** | 3/178 (1.7%) |
| **Animal protein consumption** | 75/77 (97%) |
| **Living alone** | 20/36 (56%) |
| **Care location:** *Hospitalization* | 143/177 (81%) |
| *outpatient clinic* | 34/177 (19%) |
| **Upper limb muscle weakness:** *0* | 1/172 (0.6%) |
| *2* | 1/172 (0.6%) |
| *3* | 9/172 (5.2%) |
| *4* | 32/172 (19%) |
| *5* | 129/172 (75%) |
| **Lower limb muscle weakness:** *0* | 6/167 (3.6%) |
| *1* | 3/167 (1.8%) |
| *2* | 18/167 (11%) |
| *3* | 39/167 (23%) |
| *4* | 49/167 (29%) |
| *5* | 52/167 (31%) |
| **Pyramidal signs** | 27/168 (16%) |
| **Ataxia** | 37/178 (21%) |
| **Paresthesias** | 33/178 (19%) |
| **Gait disorder** | 144/172 (84%) |
| **Rankin scale:** *0* | 4/128 (3.1%) |
| *1* | 24/128 (19%) |
| *2* | 45/128 (35%) |
| *3* | 13/128 (10%) |
| *4* | 37/128 (29%) |
| *5* | 5/128 (3.9%) |
| **Recovery (after 1 month) :** *None* | 4/64 (6.3%) |
| *Partial* | 55/64 (86%) |
| *Full* | 5/64 (7.8%) |
| **Maintained withdrawal** | 62/75 (83%) |
| **Cartridges** | 57/127 (45%) |
| **Carboys** | 91/127 (72%) |
| **Cervical MRI** | 31/33 (94%) |
| **Thoracic MRI** | 9/25 (36%) |
| **Lumbar MRI** | 0/25 (0%) |
| **MRI T1:** *Abnormal* | 15/83 (18%) |
| *~~Normal~~* | ~~68/83 (82%)~~ |
| **MRI T2:** *Abnormal* | 89/134 (66%) |
| *~~Normal~~* | ~~45/134 (34%)~~ |
| **Electromyography:**  *Axonal lesion* | 83/142 (58%) |
| *Demyelinating lesion* | 9/142 (6.2%) |
| *Mixed lesion* | 28/142 (20%) |
| *Normal* | 22/142 (15%) |
| **Protein levels in CSF (g/L)** **^1^** | 0.36 (0.18) |
| **Homocysteine** **abnormal** | 112/117 (96%) |
| **Methylmalonic acid abnormal** | 40/44 (91%) |
| **B12 treatment** | 168/176 (96%) |
| **Treatment method:** *Intra-Muscular* | 53/144 (37%) |
| *Mixed* | 32/144 (22%) |
| *Oral* | 59/144 (41%) |
| **B12 blood level before treatment** **(pg/mL)** **^1^** | 237 (111) |
| **B12 blood level after treatment** **(pg/mL)** **^1^** | 986 (806) |
| **Self-medication (oral B12)** | 18/161 (11%) |
| **Intravenous immunoglobulin** | 12 (6.8%) |
| **Corticosteroids** | 5 (2.9%) |
| ^1^n/N (%); Mean (SD) |  |

# Supplementary Figures

**Figure S1. Number of cases per hospital**

**
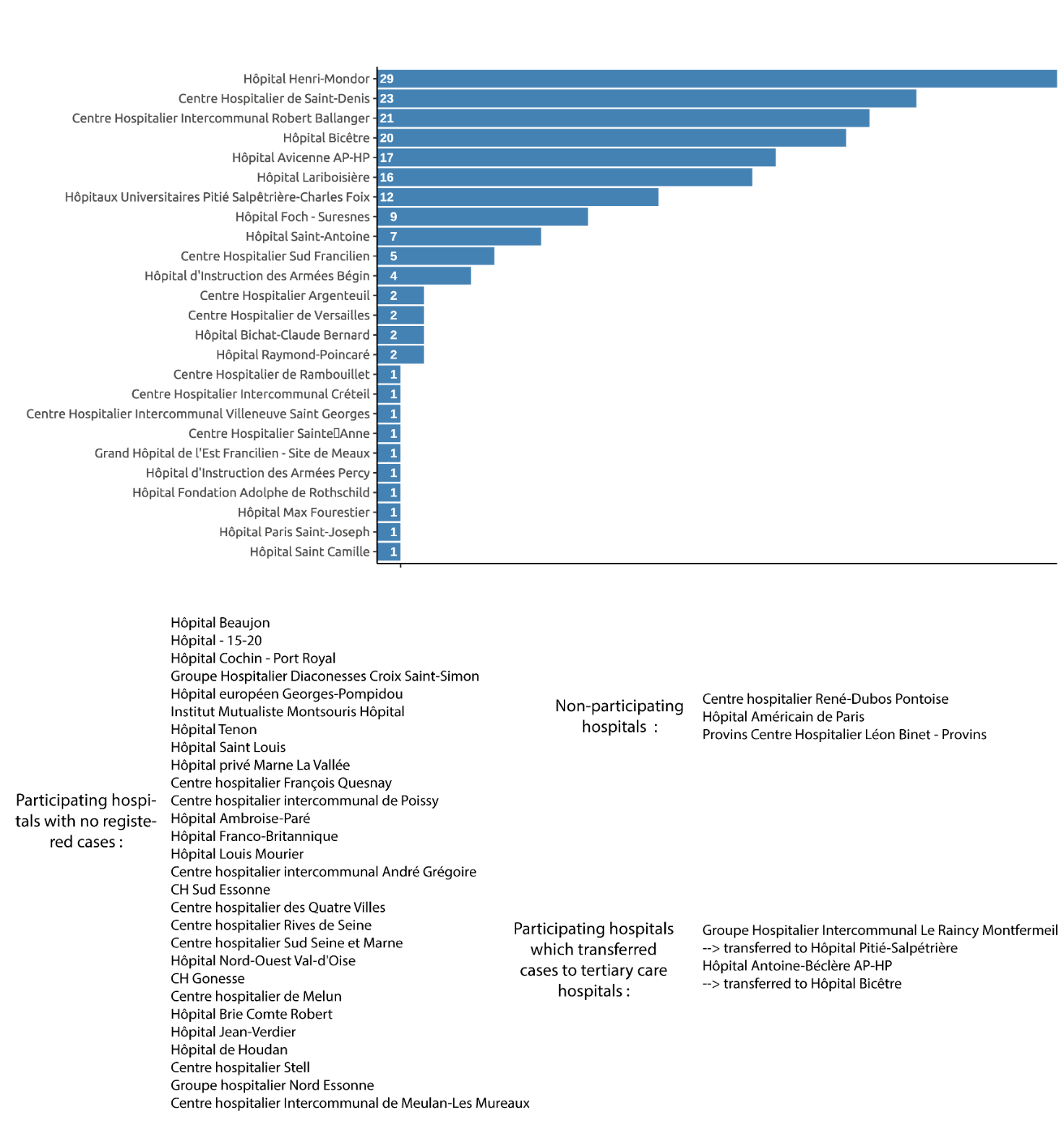
**

**Figure S2. Cases of neurological disorders linked to N_2_O in the Greater Paris area by department.** The majority of cases are located in urban areas: Paris and the inner suburbs.


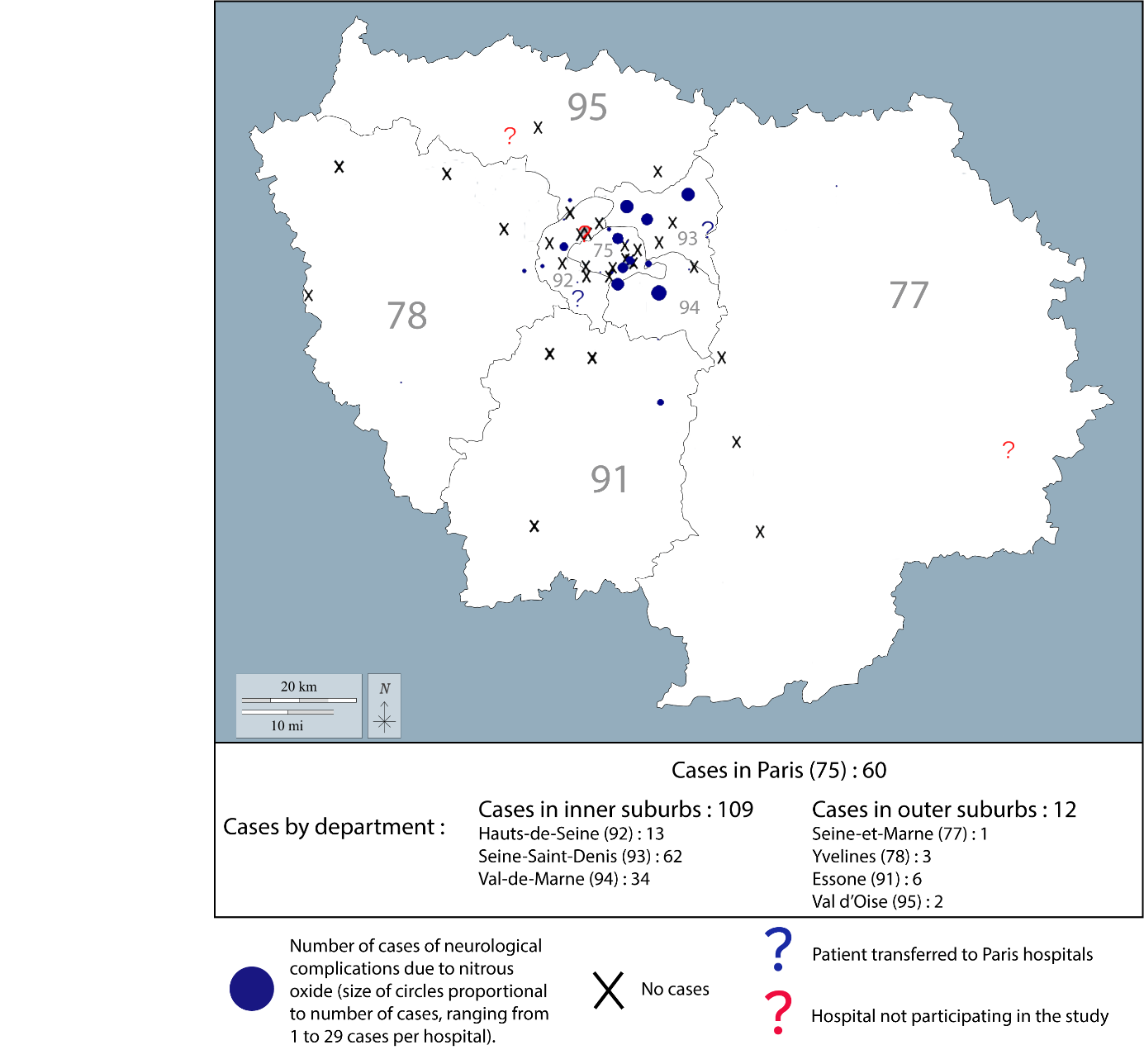

Supplement: Supplementary file 1 — Supplementary file1 (DOCX 1195 KB) [file 415_2024_12264_MOESM1_ESM.docx]
